# Acute retrobulbar optic neuritis with anti-myelin oligodendrocyte glycoprotein antibody-associated disease complicated with microscopic polyangiitis: A case report

**DOI:** 10.1097/MD.0000000000024889

**Published:** 2021-04-16

**Authors:** Tomoyuki Asano, Yuzuka Saito, Naoki Matsuoka, Jumpei Temmoku, Yuya Fujita, Kasumi Hattori, Shunsuke Kobayashi, Akira Ojima, Toshiyuki Takahashi, Haruki Matsumoto, Makiko Yashiro-Furuya, Shuzo Sato, Hiroko Kobayashi, Hiroshi Watanabe, Kiori Yano, Tomomi Sasajima, Kazuo Fujihara, Kiyoshi Migita

**Affiliations:** a Department of Rheumatology; b Department of Neurology, Fukushima Medical University School of Medicine, Fukushima; c Department of Neurology, Teikyo University School of Medicine, Tokyo; d Department of Ophthalmology, Fukushima Medical University School of Medicine, Fukushima; e Department of Neurology, Tohoku University Graduate School of Medicine, Sendai; f Department of Neurology, National Hospital Organization Yonezawa Hospital, Yonezawa; g Department of Rheumatology, Fukushima Rosai Hospital; h Department of Multiple Sclerosis Therapeutics, Fukushima Medical University School of Medicine; i Multiple Sclerosis and Neuromyelitis Optica Center, Southern Tohoku Research Institute for Neuroscience, Fukushima, Japan.

**Keywords:** anti-MOG antibodies, microscopic polyangiitis, anti-myeloperoxidase antineutrophil cytoplasmic antibody, retrobulbar optic neuritis

## Abstract

**Rationale:**

Anti-myelin oligodendrocyte protein antibody-associated disease (MOGAD) is a new disease entity with various clinical phenotypes. MOGAD often present with recurrent optic neuritis (ON), and it can also develop as a compartment of neuromyelitis optica spectrum disorder (NMOSD). Moreover, multiple autoantibodies such as an anti-myeloperoxidase antineutrophil cytoplasmic antibody (MPO-ANCA) had been reported in the serum of patients with NMOSD.

**Patient concerns:**

We report an 86-year-old woman with a 2-year history of microscopic polyangiitis (MPA). The patient had a rapid loss of vision in her left eye. No abnormal findings were observed on her left fundus, and she tested negative for MPO-ANCA upon admission. However, anti-MOG antibodies were observed in the patient's serum and cerebrospinal fluid.

**Diagnosis:**

A diagnosis of MOGAD complicated with MPA was made.

**Interventions:**

The patient received twice steroid pulse therapy and oral azathioprine as maintenance therapy.

**Outcomes:**

Her vision rapidly recovered, and no subsequent relapse was observed during the 8-month observation period.

**Conclusion:**

To the best of our knowledge, this is the first case of MOGAD complicated with MPA, and steroid pulse therapy and azathioprine therapy were effective for ON caused by MOGAD.

## 1 Introduction

The myelin oligodendrocyte glycoprotein (MOG) is expressed on the myelin sheath's outer surface; therefore, it is likely to be targeted by autoantibodies.^[1]^ A subtype of multiple sclerosis (MS) with a clinical phenotype of optic neuromyelitis has been recognized to form a distinct entity.^[2]^ The concept of neuromyelitis optica spectrum disorder (NMOSD) was proposed based on the revised international diagnostic standard criteria in 2015.^[3]^ Patients were then diagnosed with NMOSD if they test positive for anti-aquaporin 4 (AQP4) antibodies or present with one of the major clinical signs (including optic neuritis, myelitis, and brain disorders).^[3]^ In some cases, optic neuritis (ON) with positivity to anti-MOG antibody and negativity to an anti-AQP4 antibody is a disease concept that also meets the NMOSD criteria if they have one of the major clinical signs. However, in some cases that have not met NMOSD criteria, ON is considered to be caused by anti-MOG antibody-associated disease (MOGAD) independent of NMO or MS.

Interestingly, patients with NMO rarely present with positivity to myeloperoxidase antineutrophil cytoplasmic antibodies (MPO-ANCA) has been reported.^[4]^ MPO-ANCA is an autoantibody to the cytoplasm of neutrophils, and it is often positive in the serum of patients with MPA. However, there has been no report of microscopic polyangiitis (MPA) complicated with MOGAD. Herein, we present a case of retrobulbar ON of MOGAD complicated with MPA, which was successfully treated with glucocorticoid and immunosuppressants.

## 2 Case report

**Figure 1:**
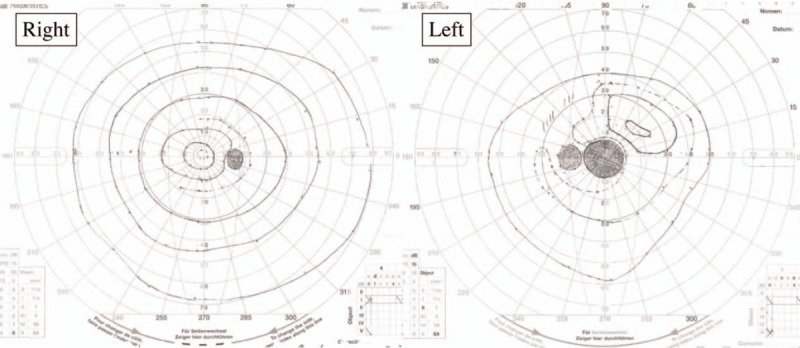
Findings in Goldman visual field test. General stenosis and a central dark spot in the left eye were shown.

**Figure 2:**
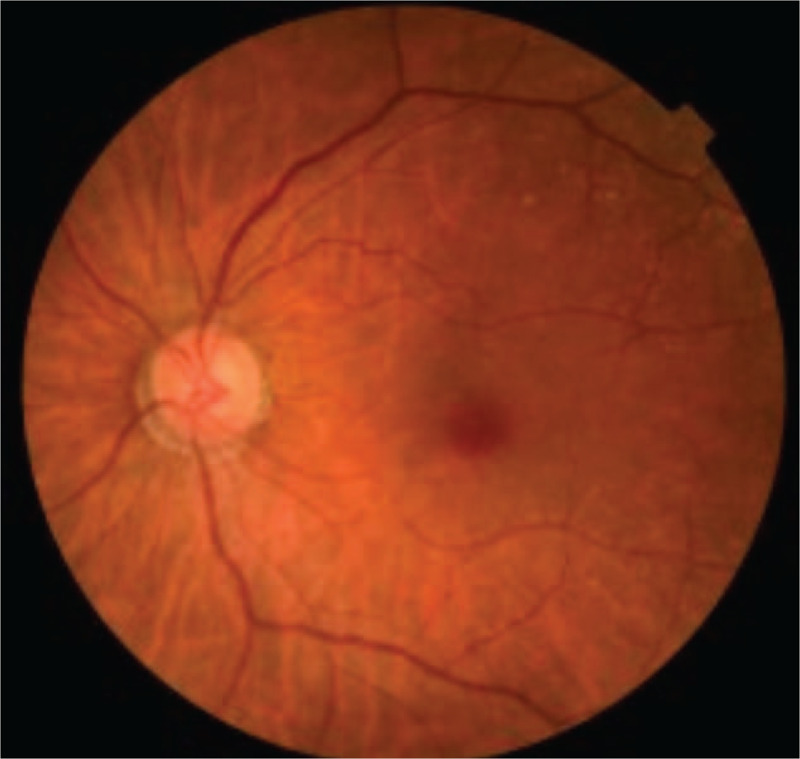
Findings in left eyeground examination. There were no abnormal findings, such as optic disk edema, pallor, or atrophy.

**Figure 3:**
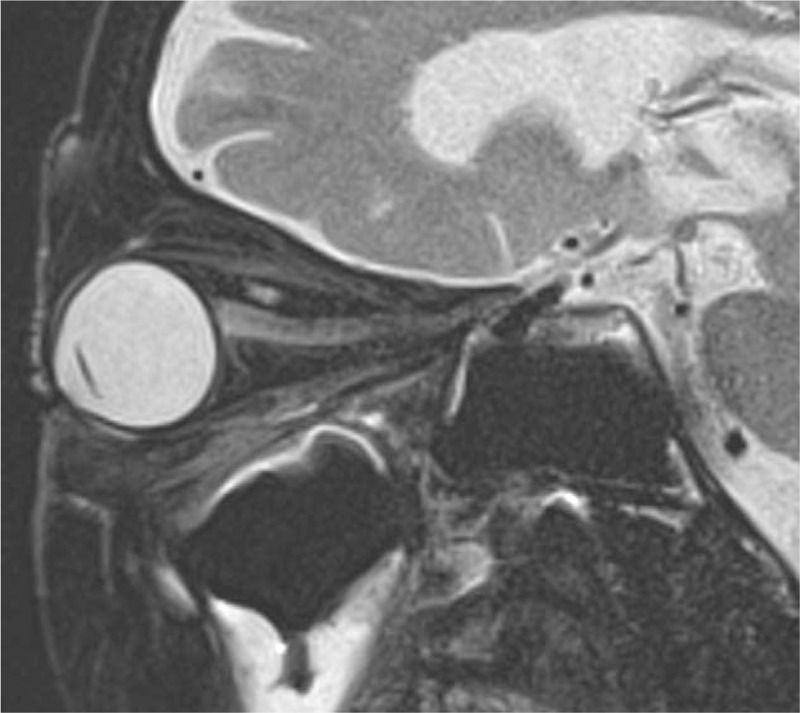
Gadolinium-enhanced magnetic resonance imaging (MRI) in the optic nerve area. There were no swelling, signal changes, nor staining in both optic nerves.

An 86-year-old woman was referred to our department due to a history of rapid blurring of vision within two weeks. In 2017, she was diagnosed with MPA due to otitis media, interstitial pneumonia, and positivity to myeloperoxidase antineutrophil cytoplasmic antibodies (MPO-ANCA) at 17.7 U/ml (<3.5 U/ml). Currently, the patient tested negative for peroxidase 3-ANCA. After providing 30 mg/day of oral prednisolone (PSL) therapy, the patient's symptoms improved, and the PSL dose was gradually reduced (9 mg/day). In April 2019, the patient presented with a blurring of vision in her left eye and eventually visited an ophthalmologist. Her left visual acuity decreased from 1.2 to 0.03. However, her cranial magnetic resonance imaging showed no organic lesions. Since ON was suspected, she was referred to the department of Ophthalmology in our hospital. Goldman's visual field test revealed general stenosis and a central dark spot in the left eye (Fig. 1). And left eyeground examination showed no abnormal findings, such as optic disk edema, pallor, and atrophy (Fig. 2). Gadolinium-enhanced magnetic resonance imaging (MRI) showed no swelling, signal changes, and staining in both optic nerves (Fig. 3). MRI of the cervical spinal cord showed no apparent abnormalities.

Laboratory data revealed a slightly elevated C-reactive protein level at 1.77 mg/dl and an increased erythrocyte sedimentation rate at 22 mm within 1 hour. The white blood cell count was normal at 7700/μl, but the neutrophil was slightly increased to 6,237/μl (81%). Previously, chronic renal dysfunction had been noted for over a year for her. However, these abnormalities were assessed due to chronic renal sclerosis rather than MPA-induced nephritis. No abnormal urinary sediment was found. The MPO-/PR3-ANCA levels were below the detection sensitivity. Cerebrospinal fluid (CSF) examination revealed a high CSF-protein level at 148 mg/dl and mild pleocytosis at 9/μl. Furthermore, the IgG index was relatively high at 1.86 (normal range: 0–0.73), and the patient tested positive for oligoclonal bands but negative for myelin basic protein and anti-AQP4 antibodies. These data indicated some inflammatory reactions in the central nervous system (CNS). The serum and CSF samples were analyzed to detect anti-myelin oligodendrocyte (MOG) antibodies using cell-based assay.^[5]^ The patient tested positive for anti-MOG antibody in the serum and CSF (1:128 and 1:4, respectively). Since our patient did not precisely fulfill the diagnostic criteria of NMOSD,^[3]^ she had diagnosed with retrobulbar ON caused by MOGAD.

Intravenous methylprednisolone (mPSL) (1.0 g/day) was administered, followed by oral PSL at a dose of 10 mg/day. Also, oral aspirin was added to prevent arterial thrombosis. Three days after intravenous mPSL therapy, her visual acuity significantly increased from 0.03 to 1.0. However, 2 weeks after the first therapy, her visual acuity decreased to 0.7, which indicated the relapse of retrobulbar ON. Thus, secondary intravenous mPSL was administered, and the dose of oral PSL was increased to 20 mg/day. Nine days after the second mPSL pulse therapy, her visual acuity improved from 0.7 to 1.2. The dose of azathioprine was increased from 50 to 100 mg/day for maintenance therapy to prevent the recurrence of retrobulbar ON, and the amount of PSL dose was gradually decreased. She was transferred to another hospital, and there was no recurrence during the 10-month observation period.

## 3 Discussion

Herein, we present a case of MPO-ANCA and anti-MOG antibody-positive retrobulbar ON, and glucocorticoid therapy was effective in improving visual acuity. The patient was diagnosed with MOGAD. Her visual acuity improved to normal levels, and there was no relapse of retrobulbar ON 10 months after receiving twice steroid pulse and oral azathioprine therapy.

MOG is a constituent protein of one type of oligodendrocytes in the neuroglia. The association between anti-MOG antibody and inflammatory demyelinating diseases of the CNS, such as NMO,^[5,6]^ and acute disseminated encephalomyelitis (ADEM),^[7]^ has been reported. In 2015, new international diagnostic criteria for NMOSD were proposed.^[3]^ According to this criterion, a diagnosis of NMOSD is made if a patient tests positive for anti-AQP4 antibody and presents with one of the major clinical signs (such as ON, myelitis, and encephalopathy).^[3]^ However, our patient did accurately meet the 2015 criteria for NMOSD.^[3]^ On the other hand, there have been many anti-MOG antibody positivity reports in cases that have symptoms similar to those of NMOSD but do not meet this criterion. By contrast, some case reports involved patients who did not satisfy this criterion and tested positive for anti-MOG antibodies and negative for anti-AQP4 antibodies.^[5,8]^ Consequently, anti-MOG antibodies were considered pathologically important.

Unlike typical NMO, MOGAD includes various types, and the most common types of disease differ depending on the age groups. It is thought that ADEM is common in childhood,^[9]^ and ON is typical in young men.^[10]^ Sequelae are not usually observed in these conditions. However, Cobo-Calvo *et al.* reported that unilateral ON and a relapsing clinical course were observed in a group of MS patients positivity to anti-MOG antibodies.^[11]^ In such cases, patients are more likely to test positive for oligoclonal bands, and MRI findings have been characterized according to paracortical and periventricular lesions similar to typical MS.^[12]^ In our case, no abnormalities were detected on MRI. However, our patient tested positive for oligoclonal bands, and we experienced relapse twice within a short period. These findings may be similar to those of MS. On the other hand, Spadaro *et al.* showed that relapsing ON cases might be similar to NMOSD because there were several cases in which various disease-modifying drugs are ineffective.^[13]^

NMOSD may be associated with other autoimmune diseases, such as systemic lupus erythematosus, Sjögren syndrome, and myasthenia gravis.^[14]^ Moreover, Long *et al.* showed that the positivity rate of perinuclear (p-) ANCA or cytoplasmic (c-) ANCA was higher in NMO than in MS, and spinal cord lesions (mainly transverse myelitis) were associated with positivity to ANCA.^[4]^ Furthermore, Gkaniatsou *et al.* reported that autoimmune thyroiditis's coexistence was observed in 6.3% of MOG-IgG seropositive cases, and 4 (33.3%) of 12 patients tested positive for antinuclear antibodies.^[15]^ In that report, MOG-IgG-positive patients tested negative for both p-ANCA and c-ANCA; however, since only 5 of 16 ANCA cases were assessed, the information obtained might be considered reference data. Besides, none of the ANCA-positive patients developed ANCA-associated vasculitis in this report. There has been no report of anti-MOG antibody-positive MPA. This is the first case report with MOGAD complicated with MPA, but the questions about the pathogenicity of ANCA in MOGAD have been raised.

Guillevin *et al.* showed that about only 1.2% of MPA patients have ocular complications.^[16]^ Regarding the ocular findings in MPA, eyelid skin nodules, conjunctival hyperemia, peripheral keratitis, episcleritis, uveitis, optic disc edema, retinal detachment, and cotton-like vitiligo have been reported.^[16–20]^ The ocular manifestations of MPA are mainly attributed to the mechanisms of small vessel necrotizing vasculitis. Thus, abnormal findings are often observed in the fundus.^[17]^ However, there were no abnormalities of the fundus in our case. Hence, ON caused by the typical mechanism of necrotizing vasculitis was ruled out. Moreover, anti-MOG antibody-positive ON was reported as unilateral or bilateral papillitis or papilloedema in 15 of 50 patients (30%), and optic disc atrophy was observed in 13 patients (59.1%).^[21]^ Furthermore, most patients with MOG antibody-positive ON were aware of posterior bulbar pain,^[21]^ which indicates a symptom of retrobulbar ON. Because coexistence with MPA was observed in our case, the mechanism of autoantibody production might have been boosted by the activation of B cells in the CNS, thereby possibly contributing to the development and progression of retrobulbar ON. Baba *et al.* reported an anti-MOG antibody-positive patient resistant to immunosuppressive therapy for primary CNS vasculitis identified via brain biopsy.^[22]^ In that report, the pathological finding in the brain tissue showed perivascular lymphocyte infiltration without demyelination, which may indicate the immunological pathogenicity of anti-MOG antibodies to the blood vessels in the CNS.^[22]^

In conclusion, whether anti-MOG antibodies and ANCA are involved in the development of ON with MOGAD remains unclear. However, predisposition to the activation of B cells that produce autoantibodies may exacerbate these pathologies. When clinicians encounter a patient with ON complicated with vasculitis, serum anti-AQP4 antibodies, and anti-MOG antibodies should be assessed, which might help obtain an accurate diagnosis.

## Acknowledgments

The authors would like to thank Toshiyuki Takahashi for his contribution to the measurement of anti-MOG antibodies. We also would like to thank Enago Group (www.enago.jp) for the English language review.

## Author contributions

**Conceptualization:** Tomoyuki Asano, Kazuo Fujihara, Kiyoshi Migita.

**Data curation:** Tomoyuki Asano.

**Formal analysis:** Tomoyuki Asano.

**Funding acquisition:** Tomoyuki Asano.

**Investigation:** Tomoyuki Asano, Yuzuka Saito, Naoki Matsuoka, Jumpei Temmoku, Yuya Fujita, Kasumi Hattori, Shunsuke Kobayashi, Akira Ojima, Haruki Matsumoto, Makiko Yashiro-Furuya, Shuzo Sato, Hiroko Kobayashi, Hiroshi Watanabe, Kiori Yano, Tomomi Sasajima, Kiyoshi Migita.

**Methodology:** Tomoyuki Asano, Yuzuka Saito, Naoki Matsuoka, Jumpei Temmoku, Yuya Fujita, Kasumi Hattori, Shunsuke Kobayashi, Akira Ojima, Toshiyuki Takahashi, Haruki Matsumoto, Makiko Yashiro-Furuya, Shuzo Sato, Hiroko Kobayashi, Hiroshi Watanabe, Kiori Yano, Tomomi Sasajima, Kiyoshi Migita.

**Project administration:** Tomoyuki Asano, Yuzuka Saito, Naoki Matsuoka, Jumpei Temmoku, Yuya Fujita.

**Resources:** Tomoyuki Asano, Yuzuka Saito, Naoki Matsuoka, Jumpei Temmoku, Yuya Fujita, Akira Ojima, Toshiyuki Takahashi.

**Supervision:** Hiroshi Watanabe, Kazuo Fujihara, Kiyoshi Migita.

**Validation:** Tomoyuki Asano, Kiyoshi Migita.

**Visualization:** Tomoyuki Asano.

**Writing – original draft:** Tomoyuki Asano.

**Writing – review & editing:** Tomoyuki Asano.
